# A set of microsatellite markers to differentiate *Plasmodium falciparum* progeny of four genetic crosses

**DOI:** 10.1186/s12936-018-2210-z

**Published:** 2018-02-02

**Authors:** Christine E. Figan, Juliana M. Sá, Jianbing Mu, Viviana A. Melendez-Muniz, Chia Hao Liu, Thomas E. Wellems

**Affiliations:** Laboratory of Malaria and Vector Research (LMVR), National Institute of Allergy and Infectious Diseases (NIAID), National Institutes of Health (NIH), Bethesda, MD 20892 USA

**Keywords:** *Plasmodium falciparum*, Genotyping, Molecular fingerprint, Parasite clones, Cross-contamination

## Abstract

**Background:**

Four *Plasmodium falciparum* genetic crosses (HB3×3D7, HB3×Dd2, 7G8×GB4, and 803×GB4) have produced sets of recombinant progeny that are widely used for malaria research, including investigations of anti-malarial drug resistance. It is critical to maintain the progeny free from cross-contamination. Microsatellite polymorphisms can be used to validate parasite identity.

**Results:**

A set of 12 markers was developed that differentiates the parents of the four *P. falciparum* crosses. This typing set identified distinguishing patterns of inheritance (fingerprints) in segregant collections of 15 progeny clones from HB3×3D7, 32 from HB3×Dd2, 33 from 7G8×GB4, and 81 from 803×GB4. Stronger amplification was observed with shorter relative to longer alleles of individual microsatellites. In experiments with mixed parental DNAs, electropherograms showed that signals of cross-contamination can be missed when minor peaks less than 1/4 or 1/3 the height of the major peak are disregarded by threshold settings commonly used for population studies.

**Conclusions:**

Microsatellite typing is an effective method to check the identity of *P. falciparum* lines and detect parasite cross-contamination in cultures; however, care must be taken not to ignore minor peaks that can be overlooked. The 12 microsatellite markers presented here provide a rapid and efficient means to distinguish the segregants of laboratory crosses. Fingerprint patterns from these markers are useful to maintain the integrity of diverse parasite lines in and between research laboratories.

**Electronic supplementary material:**

The online version of this article (10.1186/s12936-018-2210-z) contains supplementary material, which is available to authorized users.

## Background

Genetic crosses between *Plasmodium falciparum* lines with distinct phenotypes have supported the identification of genes involved in malaria drug resistance, immune evasion, host infection, and disease pathogenesis. However, reproducing the malaria parasite life cycle in the laboratory is complex and expensive [1]. The process requires: production of infectious gametocytes from parasite cultures; infection of *Anopheles* mosquitoes with these gametocytes for cross-fertilization and generation of recombinant sporozoites (*Plasmodium* are haploid except for a brief diploid phase from zygote to oocyst); inoculation of the sporozoites into a vertebrate host for production of liver stage parasites; and recovery of blood-stage recombinant progeny (segregants) that emerge from the liver and propagate in erythrocytes. Five *P. falciparum* genetic crosses have been completed over the past 40 years. Four of these crosses were performed through chimpanzees (Table 1) [2–4] (and unpublished work). More recently, a fifth cross was produced through immune-deficient mice engrafted with liver and erythrocytes from humans [5].

**Table 1:** Four *Plasmodium falciparum* genetic crosses in nonhuman primates

	3D7×HB3	HB3×Dd2	7G8×GB4	GB4×803
Year published	1987 (Walliker et al.)	1990 (Wellems et al.)	2008 (Hayton et al.)	Unpublished
Parental origins	3D7: Netherlands HB3: Honduras	HB3: Honduras Dd2: Indochina	7G8: Brazil GB4: Ghana	GB4: Ghana 803: Cambodia
Typing method	Restriction fragment length polymorphism (RFLP)	RFLP and microsatellite analysis	Microsatellite analysis and DNA microarray	Microsatellite analysis and DNA microarray
Potential segregants	15	32	33	81

Recombinant progeny from the four non-human primate (NHP) crosses have led to fundamental research advances in diverse studies of malaria phenotypes (Additional file 1). Protecting the integrity of the *P. falciparum* parent and progeny lines has been critical to their value as a research resource in support of these and other advances. In a laboratory setting where multiple culture flasks are handled, investigators must continually guard against the risks of cross-contamination or mislabeling [6, 7]. Vigilance is imperative, and it is necessary to perform regular checks of parasites recovered from cryopreserved vials, in long-term cultures, and before cryopreservation for legacy stocks.

Progeny of the *P. falciparum* genetic crosses have been typed by various methods. HB3×3D7 progeny were originally typed by restriction fragment length polymorphism (RFLP) methods in which parasite DNA cut by restriction enzymes is separated on agarose gels, blotted to a membrane, and probed for DNA fragment size differences [8, 9]. HB3×Dd2 progeny were typed by RFLP [10] and microsatellite analysis [11, 12], a method that utilizes polymerase chain reaction (PCR) to amplify stretches of short polymorphic repeats that can be highly variable between parasite lines. Microsatellite and tandem repeat regions that differ by many base pairs can be visualized on agarose gels, whereas microsatellite regions that differ slightly in size are more appropriately analyzed by polyacrylamide sequencing gel or capillary electrophoresis. 7G8×GB4 progeny were typed using microsatellites and high-density oligonucleotide tiling arrays, which differentially hybridize with fragments of DNA containing single nucleotide polymorphisms (SNPs) [13, 14]. 803×GB4 progeny have been typed using microsatellites and a DNA microarray containing 17,000 potential SNPs (unpublished). More recently, progeny from the HB3×3D7, HB3×Dd2, and 7G8×GB4 crosses were analyzed by full genome sequencing [15].

A typing method must be fast, reliable, and inexpensive for regular checks of parasite cultures in the laboratory. RFLP methods require large amounts of DNA, and probe preparation and blotting after gel electrophoresis is time consuming. DNA microarrays also require large amounts of DNA and depend on skilled processing and analysis with specialized equipment. Modern genome sequencing can give snapshots of total variation, but cost, turnaround time, and analysis remain prohibitive for routine typing checks. In contrast to these methods, microsatellite analysis has a production time of less than 1 day, requires only small amounts of DNA, and is relatively inexpensive [12].

A set of 12 microsatellite markers that distinguishes progeny of the four NHP *P. falciparum* crosses is presented here. Use of these markers can be extended to diverse parasite lines and help to maintain their integrity and identity among different laboratories.

## Methods

### Parasite cultivation and genomic DNA

Erythrocytes obtained weekly from Interstate Blood Bank (Memphis, TN) or Virginia Blood Services (Richmond, VA) were washed with filtered RPMI 1640 media (KD Medical, Columbia, MD) and stored at 50% hematocrit in a 4 °C refrigerator until use. Complete culture media (cRPMI) was comprised of 1% Albumax II (Life Technologies, Carlsbad, CA), 0.21% sodium bicarbonate (KD Medical, Columbia, MD), and 20 µg/mL gentamicin (KD Medical, Columbia, MD) in RPMI-1640, which contains 25 mM HEPES and 50 µg/mL hypoxanthine. Parasites were cultivated at 5% haematocrit between 0.5–3.0% parasitaemia at 37 °C under an atmosphere of 90% N_2_, 5% CO_2_, and 5% O_2_. When cultures reached approximately 4% parasitaemia with mostly mature stages, 10 mL volumes were pelleted at 2500 rpm, and the parasitized cells were treated with 0.15% saponin (Amresco, Solon, Ohio) in 1× phosphate buffer solution (PBS) (90 g/L sodium chloride, 1.44 g KH_2_PO_4_/L, 7.95 g NA_2_HPO_4_/L) (KD Medical, Columbia, MD) for 3 min at room temperature. Parasites were recovered from the resulting host cell lysate by centrifugation at 4000 rpm, washed three times with 1× PBS, and stored at − 20 °C. Some samples of genomic DNA from the 803×GB4 progeny were obtained from smaller culture volumes (~ 2 mL). Genomic DNA was extracted from thawed samples by the phenol:chloroform method [16] or by the DNeasy Blood & Tissue Kit (Qiagen, Hilden, Germany). DNA concentration was determined using a NanoDrop ND-100 Spectrophotometer.

### Polymerase chain reaction

PCR was performed in 96-well plates in an Applied Biosystems SimpliAmp Thermal Cycler with the following conditions: denaturation for 2 min at 94 °C; 42 cycles through 94 °C for 20 s, 45 °C for 10 s, 42 °C for 10 s, and 60 °C for 30 s; and a final extension at 60 °C for 5 min. Reaction volumes of 25 µL included: 1 µL of template DNA at a concentration of approximately 20 ng/µL, 0.5 µL of each 10 µM forward primer and 10 µM reverse primer with a 5′ 6-FAM fluorescence modification (Eurofins, Louisville, KY), 12.5 µL MyTaq Mix (Bioline, Taunton, MA), and 10.5 µL PCR qualified water (Quality Biological, Inc., Gaithersburg, MD).

### Microsatellite analysis

Amplified DNA was diluted 100× in an optical 96-well plate with 10 µL of Hi-Di Formamide (Life Technologies Corporation, Carlsbad, CA) and 0.2 µL GeneScan 500XL ROX Size Standards (Life Technologies Corporation, Carlsbad, CA) per well. Plates were processed in a Hitachi 3730xl DNA Analyzer and evaluated using GeneMapper version 4.1 (Applied Biosystems, Foster City, CA). Base pair sizes of microsatellite alleles were recorded at the peak heights of their signals presented on electropherograms.

### Mixed DNA samples experiment

Stock 803 and GB4 genomic DNA dilutions were combined in 1.5 mL Eppendorf tubes (Denville Scientific Incorporated, Holliston, MA) in 803:GB4 DNA ratios of 5:95, 10:90, 20:80, 50:50, 80:20, 90:10, and 95:5. These mixed DNA samples were typed as described above using markers TAA87, TA127, 1451458, and C3M69.

## Results and discussion

Thirty-two candidate microsatellite markers were identified from a large collection originally developed to differentiate HB3×Dd2 and 7G8×GB4 progeny (Table 2 and Additional file 2) [4, 11]. Selection criteria included: (1) physical location in the genome to avoid linkage disequilibrium and cover the most chromosomes; and (2) PCR product size that discriminated between the parents of each cross by more than 3 base pairs. Of these 32 microsatellite markers, 14 distinguished the 3D7, Dd2, HB3, 7G8, GB4, and 803 parental lines from one another. To facilitate efficient PCR in the 12-column format of 96-well plates, a final set of 12 markers was selected for typing. Table 2 lists the primer sequences, genome locations, and expected PCR product sizes of the 12 microsatellites.

**Table 2:** A set of 12 microsatellite markers that distinguishes parents and recombinant progeny of four *Plasmodium falciparum* crosses

Marker	Chromosome	Location (kB)	Microsatellite peak size (base pairs)						Forward primer	Reverse primer	Microsatellite repeat sequence-including F/R primers (3D7)
			GB4	803	Dd2	HB3	3D7	7G8			
C3M64	3	477	109	174	174	184	198	188	AAAGAATAAGAATAGGAATCA	AACAAAACATGCTTATCTAAA	AAAGAATAAGAATAGGAATCATATATATATACATACATATACACACATATATATATATATATACATATATATATACACACATATATATATATACACATATATATATATATATATATATATATATATATATATATATATATATATATATATACATATTAATAAAGATTCATTTTATATTATTTAGATAAGCATGTTTTGTT
C3M54	3	909	215	264	234	241	238	266	AATATAATCATAAAGTCGTAC	CTAAGAGAAAAAATGGGTAT	AATATAATCATAAAGTCGTACTTTTTTTTTTTTATTTTATATAATAAATAAATATAAAATAAATAAAATATTTATATATATATATATATATATAAATGTTATTATATATATATATATATATATATATATATATATATTTTTATAATATATATAATAACTGGATTCTATTTTTTTTTAGAGTAATATATATATATATATATATAATTATATTTTTATATAATACCCATTTTTTCTCTTAG
C4M30	4	1087	164	189	165	180	193	167	ATTGATGCTTTGTCTAATTAG	ATGACAAAACATGGTATGTA	ATTGATGCTTTGTCTAATTAGAGTATATGATATATATATATATATAAAAAAAAAAAAATAAAAAATAAAATAAATAAAAAATAAAAAAAATAAAAAATAAAAAATAAAATATATATATATATATATATATATATATATATATATATATATATATTTTTTTTTATATATTCATATACATACCATGTTTTGTCAT
B5M3	5	494	120	114	123	127	121	133	AAGTTATGATGAATTTGATTC	TTATTATCATCATCACCCATC	AAGTTATGATGAATTTGATTCTAAAATATCAAAATATCCAACAAAAGTAGGAAATGAAGGGAAAAAATCAAAAGATAATAATAATAATAATAATAATAATATGATGGGTGATGATGATAATAA
TAA81	5	1214	121	115	121	127	118	115	TTTCACACAACACAGGATT	TGGACAAATGGGAAAGGATA	TTTCACACAACACAGGATTATTACCATCACCATTAATGTTATCATTCCTTGTATTATTATTATTATTATTATTATTATTATGTTTGATTTGGTTATCATCTTTATCCTTTCCCATTTGTCCA
TAA87	6	374	94	109	107	104	95	110	ACATGTTCATATTACTCAC	AATGGCAACACCATTCAAC	ACATGTTCATATTACTCACCACATTATTATTATTATTATTATTGTTGTTGTTGTTGTTGTTGTTGTTGTTGTTGTTAGGTGGTTGAATGGTGTTGCCATT
PE14D	7	413	114	130	115	133	136	121	TGTAATGAATGATTCTAATACCAC	TTGGACCATGCTTCACAG	TGTAATGAATGATTCTAATACCACTACTACTACTACTACTACTACTACTACTACTAATAATAATAATAATAATAATAATAATAATAATAATAATAATAAACCTACTTCCTATTTGGATTTCTGTGAAGCATGGTCCAA
TA127	8	1161	118	132	132	124	120	124	GCTTTATAAAAATAACACACC	TAAAAAACACTCAGTTTGAG	GCTTTATAAAAATAACACACCTATATATAAATATATATATATATATATATATATATATTATATACATTTCTATCTTTATTCTTTCTTTTTATTTTTTTTTTTTATCTCAAACTGAGTGTTTTTTA
C9108	9	1145	96	122	113	116	137	114	AATATCTACACAACTAAAGC	CAAAAGAATCATCCTTTTCC	AATATCTACACAACTAAAGCATATACATATATATATTATATATATATATATATATATATATATATATATATATATATATATAATATATATGTATATATTTTTTTTTTTTTTTTAATAAATGGAAAAGGATGATTCTTTTG
401780	10	402	103	80	80	105	90	86	GAAACATAAAGGGATGTGTA	ACTTAGAAGAAATTCAATGC	GAAACATAAAGGGATGTGTATAAGTATATGTATATATATATATATATATATATATATATATATTTCTTATTGATCGCATTGAATTTCTTCTAAGT
1451458	14	1452	215	203	203	194	205	209	GAGCATTATAAAATTGGCTA	AAAAAGAAAAGAGATGAACA	GAGCATTATAAAATTGGCTACTAGTGAATATCTAAAATGTACATAAATAAATAAATAAATAAATAAATAAATATGTACATATATATATATATATATATATATATATCAAAATGTACAAAAATAGAATTTAAAGTATACTTTTATCTATCTGTACCAAATATGTGTTACATATTTATATATAATAATGTTCATCTCTTTTCTTTTT
2549455	14	2550	178	174	179	176	179	175	AAATATATGTTCTACTTTCAATCA	GAGGAAATAATATAATACACCA	AAATATATGTTCTACTTTCAATCATATAATAATTTTTTTTTTTTTTACTTATTAGTTTATAACTAAAAATTTTTCAATATATATATATATATATATATATATTCAGTACTCTATTAATTATAATTATTATATGTATCTATTTTTTATATTATTAATAGATGGTGTATTATATTATTTCCTC

The 12-marker typing set identified distinguishing patterns of inheritance (fingerprints) in segregant collections of 15 progeny clones from HB3×3D7, 32 from HB3×Dd2, 33 from 7G8×GB4, and 81 from the new 803×GB4 (Additional file 3). Since peaks on the DNA analyzer can shift slightly depending on machine running conditions, parental control DNAs were included in all typing determinations along with the size standards.

**Fig. 1:**
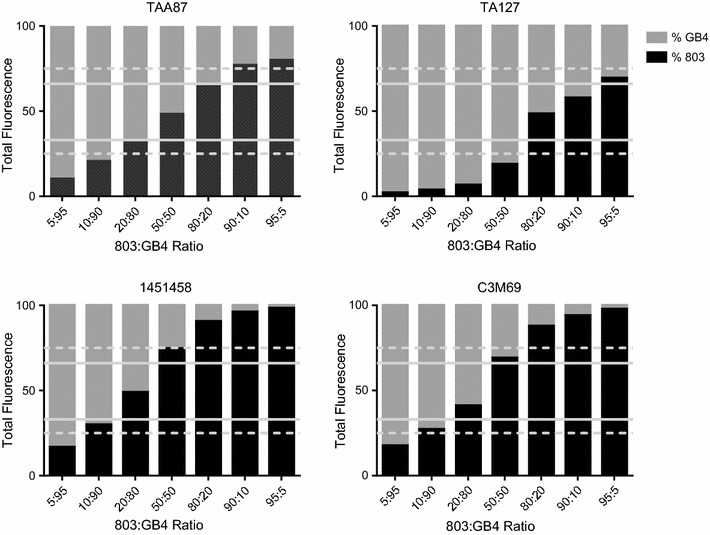
Typing results from mixtures of 803 and GB4 DNA tested with microsatellites TAA87, TA127, 1451458, and C3M69. The 803 DNA has a longer repeat sequence than GB4 in microsatellites TAA87 and TA127, whereas GB4 has a longer microsatellite sequences than 803 in microsatellites 1451458 and C3M69. Solid and dashed lines represent the 1/3 and 1/4 cut off levels, respectively

Minor mixed or contaminated parasite subpopulations can be missed by microsatellite typing when they represent < 20% of the overall parasite population [17, 18]. In further experiments with microsatellite markers to detect cross-contamination, known amounts of 803 and GB4 genomic DNA were mixed and tested with markers TAA87, TA127, 1451458, and C3M69, all of which have alleles that differ by 10 base pairs or more. The microsatellite signals from minor quantities of DNA differed greatly in level and, in some cases, were difficult to detect in the individual scans (Fig. 1). Peak height ratios were consistent with greater amplification of smaller alleles of the four individual markers, in agreement with previous findings of a general bias to smaller amplicons [18] (see representative TAA87 and TA127 data in Additional file 4).

While microsatellite analysis is an efficient method to check parasite line integrity, our results show that minor populations may still be missed. Filters that disregard minor peaks less than 1/4 or 1/3 the size of the major peak are sometimes used in population genetic studies, so the use of these filters in microsatellite analysis may give false impression of clonality with mixed parasite populations [19–22]. Unfiltered searches for minor peaks with multiple markers are therefore recommended for detection of allelic variations indicative of mixed or contaminated parasite subpopulations.

The polymorphism of these microsatellite markers can be useful for genetic population studies [19, 20, 23, 24] as well as future typing applications in genetic crosses. Although the microsatellite markers were stable in this study, infrequent de novo size variations are possible; an example has been reported in a clonal parasite population after long-term culture [11].

Comparison of sample typing patterns to published fingerprints presently relies on human ability to recognize visual patterns and find differences, and potential for error remains in assessment of clonality or disparity between lines. Automated searches may help to improve these comparisons in the future, perhaps with algorithms of pattern matching against microsatellite databases.

## Conclusions

A set of 12 microsatellite markers is presented to distinguish genetically related *P. falciparum* progeny of laboratory crosses, with useful fingerprints to maintain the integrity of diverse parasite lines in and between research laboratories.

## Additional files

**Additional file 1.** “Fundamental insights from four *P. falciparum* genetic crosses” summarizes major findings and reports using the *P. falciparum* genetic crosses.

**Additional file 2.** “Additional microsatellite markers” includes parental types from microsatellite markers other than the set of 12 presented in the main text.

**Additional file 3.** “Microsatellite fingerprints of genetic crosses” includes four worksheets of data from the individual crosses.

**Additional file 4.** “Mixed Sample Electropherograms” shows representative results from mixed DNA microsatellite typing.

## Abbreviations

PCR: polymerase chain reaction
NHP: nonhuman primate
RFLP: restriction fragment length polymorphism
SNP: single nucleotide polymorphism
cRPMI: complete RPMI 1640 media
PBS: phosphate buffer solution
