# Supplementary material for: A set of microsatellite markers to differentiate Plasmodium falciparum progeny of four genetic crosses
Source: Malar J. 2018 Feb 2;17:60. doi: 10.1186/s12936-018-2210-z (PMC5797376; doi:10.1186/s12936-018-2210-z)
Supplement: Supplementary file 1 — Additional file 1. “Fundamental insights from four P. falciparum genetic crosses” summarizes major findings and reports using the P. falciparum genetic crosses. [file 12936_2018_2210_MOESM1_ESM.docx]

Additional file 1. Fundamental scientific insights from four *P. falciparum* genetic crosses

| Genetic Cross | | Findings |
| --- | --- | --- |
| 3D7 x HB3 | - Recombinant *P. falciparum* progeny can be produced and isolated through a nonhuman primate model [1] - Fourteen chromosomes comprise the nuclear genome of *P. falciparum* [2] - Histidine-rich protein III marks a linkage group favored in the 3D7 x HB3 genetic cross [2] - A point mutation in dihydrofolate-reductase thymidylate synthase confers pyrimethamine resistance in malaria [3] | |
| HB3 x Dd2 | - Chloroquine resistance is not linked to multidrug resistance (*mdr*)-like genes [4] - Discovery of PfCRT as the major determinant of *P. falciparum* chloroquine resistance [5] - Frequent ectopic recombination occurs among telomeric clusters of *P. falciparum* *var* genes [6] - Sulfadoxine resistance is linked to mutations in dihydropteroate synthetase and a factor associated with folate utilization [7] - Multiple genes associated with low-level quinine resistance by QTL mapping [8] - *P. falciparum* male development gene 1 (*pfmdv*-1) plays a key role in gametocyte membrane formation and integrity [9] - The *pfmdr*-1 gene is linked to import of a variety of solutes into *P. falciparum* food vacuoles [10] - Polymorphic regulatory regions mediate transcriptional variations that contribute to phenotypic variation [11] - Malaria parasite *clag3* genes determine nutrient uptake by the plasmodial surface anion channel (PSAC) [12] - Description of inherited and *de novo* copy number variants (CNV) in progeny of the HB3 x Dd2 cross [13] | |
| 7G8 x GB4 | - *P. falciparum* reticulocyte binding protein homologue 5 (*PfRh5*) is key to infection of *Aotus* erythrocytes [14] - Characterization of meiotic crossover rates and recombination hotspots in *P. falciparum* chromosomes [15] - Gene *Pfs47* mediates *P. falciparum* evasion of the *Anopheles gambiae* immune system [16] | |
| GB4 x 803 | - Analysis of artemisinin resistance and K13 580Y mutation in a *P. falciparum* cross (Sa et al., unpublished) | |
| Combined analysis of the above crosses | - Major genes for differential chemical phenotypes identified in high-throughput chemical library screens [17] - Interactions between *pfcrt* and *pfmdr1* alleles mediate amodiaquine and chloroquine response phenotypes [18] - PfCRT and PfMDR1 are both transporters and targets of antimalarial drugs [19] - Indels, structural variation, and recombination drive genomic diversity in *Plasmodium falciparum* [20] | |

References:

1. Walliker D, Quakyi IA, Wellems TE, McCutchan TF, Szarfman A, London WT, et al. Genetic analysis of the human malaria parasite *Plasmodium falciparum***.** Science. 1987;236:1661-6.

2. Wellems TE, Walliker D, Smith CL, do Rosario VE, Maloy WL, Howard RJ, et al. A histidine-rich protein gene marks a linkage group favored strongly in a genetic cross of *Plasmodium falciparum***.** Cell. 1987;49:633-42.

3. Peterson DS, Walliker D, Wellems TE. Evidence that a point mutation in dihydrofolate-reductase thymidylate synthase confers resistance to pyrimethamine in falciparum malaria**.** P Natl Acad Sci USA. 1988;85:9114-8.

4. Wellems TE, Panton LJ, Gluzman IY, do Rosario VE, Gwadz RW, Walker-Jonah A, et al. Chloroquine resistance not linked to *mdr*-like genes in a *Plasmodium falciparum* cross**.** Nature. 1990;345:253-5.

5. Fidock DA, Nomura T, Talley AK, Cooper RA, Dzekunov SM, Ferdig MT, et al. Mutations in the *P. falciparum* digestive vacuole transmembrane protein PfCRT and evidence for their role in chloroquine resistance**.** Mol Cell. 2000;6:861-71.

6. Freitas-Junior LH, Bottius E, Pirrit LA, Deitsch KW, Scheidig C, Guinet F, et al. Frequent ectopic recombination of virulence factor genes in telomeric chromosome clusters of *P. falciparum***.** Nature. 2000;407:1018-22.

7. Wang P, Read M, Sims PF, Hyde JE. Sulfadoxine resistance in the human malaria parasite *Plasmodium falciparum* is determined by mutations in dihydropteroate synthetase and an additional factor associated with folate utilization**.** Mol Microbiol. 1997;23:979-86.

8. Ferdig MT, Cooper RA, Mu J, Deng B, Joy DA, Su X-z, et al. Dissecting the loci of low-level quinine resistance in malaria parasites**.** Mol Microbiol. 2004;52:985-97.

9. Furuya T, Mu J, Hayton K, Liu A, Duan J, Nkrumah L, et al. Disruption of a *Plasmodium falciparum* gene linked to male sexual development causes early arrest in gametocytogenesis**.** Proc Natl Acad Sci U S A. 2005;102:16813-8.

10. Rohrbach P, Sanchez CP, Hayton K, Friedrich O, Patel J, Sidhu AB, et al. Genetic linkage of *pfmdr1* with food vacuolar solute import in *Plasmodium falciparum***.** EMBO J. 2006;25:3000-11.

11. Gonzales JM, Patel JJ, Ponmee N, Jiang L, Tan A, Maher SP, et al. Regulatory hotspots in the malaria parasite genome dictate transcriptional variation**.** PLoS Biol. 2008;6:e238.

12. Nguitragool W, Bokhari AA, Pillai AD, Rayavara K, Sharma P, Turpin B, et al. Malaria parasite *clag3* genes determine channel-mediated nutrient uptake by infected red blood cells**.** Cell. 2011;145:665-77.

13. Samarakoon U, Gonzales JM, Patel JJ, Tan A, Checkley L, Ferdig MT. The landscape of inherited and *de novo* copy number variants in a *Plasmodium falciparum* genetic cross**.** BMC Genomics. 2011;12:457.

14. Hayton K, Gaur D, Liu A, Takahashi J, Henschen B, Singh S, et al. Erythrocyte binding protein PfRH5 polymorphisms determine species-specific pathways of *Plasmodium falciparum* invasion**.** Cell Host Microbe. 2008;4:40-51.

15. Jiang H, Li N, Gopalan V, Zilversmit MM, Varma S, Nagarajan V, et al. High recombination rates and hotspots in a *Plasmodium falciparum* genetic cross**.** Genome Biol. 2011;12:R33.

16. Molina-Cruz A, Garver LS, Alabaster A, Bangiolo L, Haile A, Winikor J, et al. The human malaria parasite Pfs47 gene mediates evasion of the mosquito immune system**.** Science. 2013;340:984-7.

17. Yuan J, Cheng KC, Johnson RL, Huang R, Pattaradilokrat S, Liu A, et al. Chemical genomic profiling for antimalarial therapies, response signatures, and molecular targets**.** Science. 2011;333:724-9.

18. Sa JM, Twu O, Hayton K, Reyes S, Fay MP, Ringwald P, et al. Geographic patterns of *Plasmodium falciparum* drug resistance distinguished by differential responses to amodiaquine and chloroquine**.** Proc Natl Acad Sci U S A. 2009;106:18883-9.

19. Sanchez CP, Mayer S, Nurhasanah A, Stein WD, Lanzer M. Genetic linkage analyses redefine the roles of PfCRT and PfMDR1 in drug accumulation and susceptibility in *Plasmodium falciparum***.** Mol Microbiol. 2011;82:865-78.

20. Miles A, Iqbal Z, Vauterin P, Pearson R, Campino S, Theron M, et al. Indels, structural variation, and recombination drive genomic diversity in *Plasmodium falciparum***.** Genome Res. 2016;26:1288-99.
