# Supplementary material for: A set of microsatellite markers to differentiate Plasmodium falciparum progeny of four genetic crosses
Source: Malar J. 2018 Feb 2;17:60. doi: 10.1186/s12936-018-2210-z (PMC5797376; doi:10.1186/s12936-018-2210-z)

Additional file 4. Mixed Sample Electropherograms

Marker	DNA ratio 803:GB4	Microsatellite Region Sizes
**TAA87**	**5:95**	**803—109bp GB4—94bp**

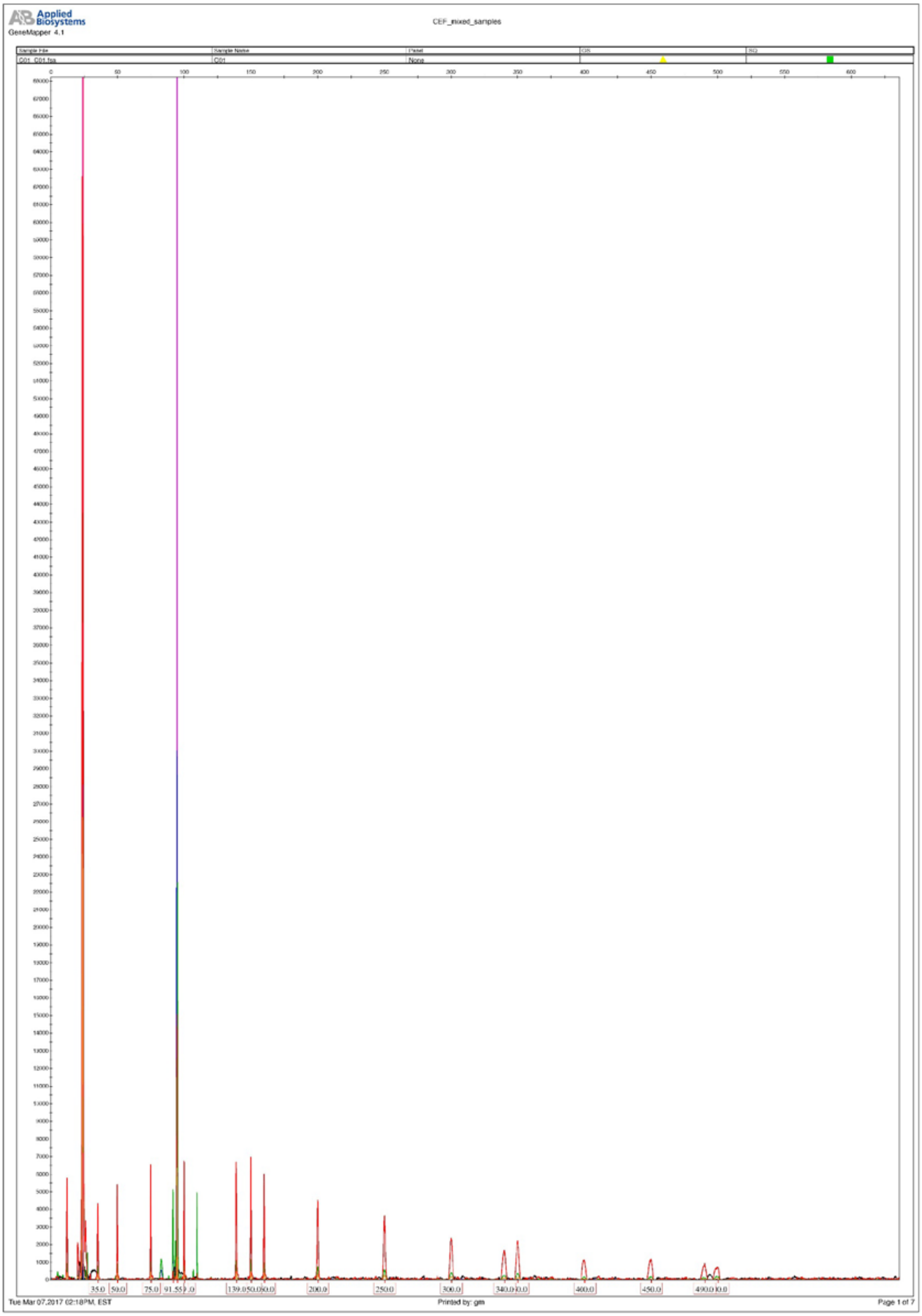

Additional file 4. Mixed Sample Electropherograms

Marker	DNA ratio 803:GB4	Microsatellite Region Sizes
**TAA87**	**10:90**	**803—109bp GB4—94bp**

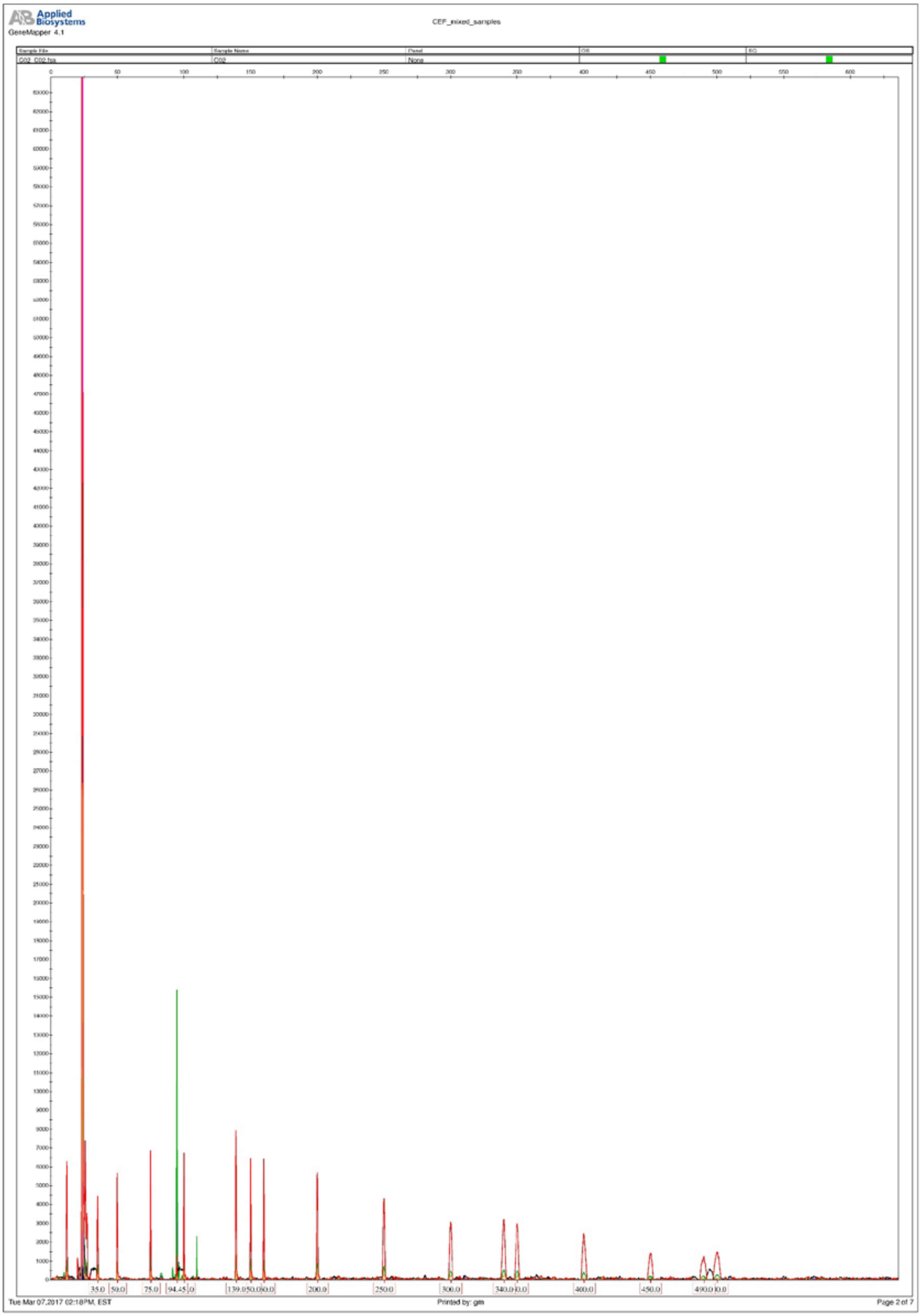

Additional file 4. Mixed Sample Electropherograms

Marker	DNA ratio 803:GB4	Microsatellite Region Sizes
**TAA87**	**20:80**	**803—109bp GB4—94bp**

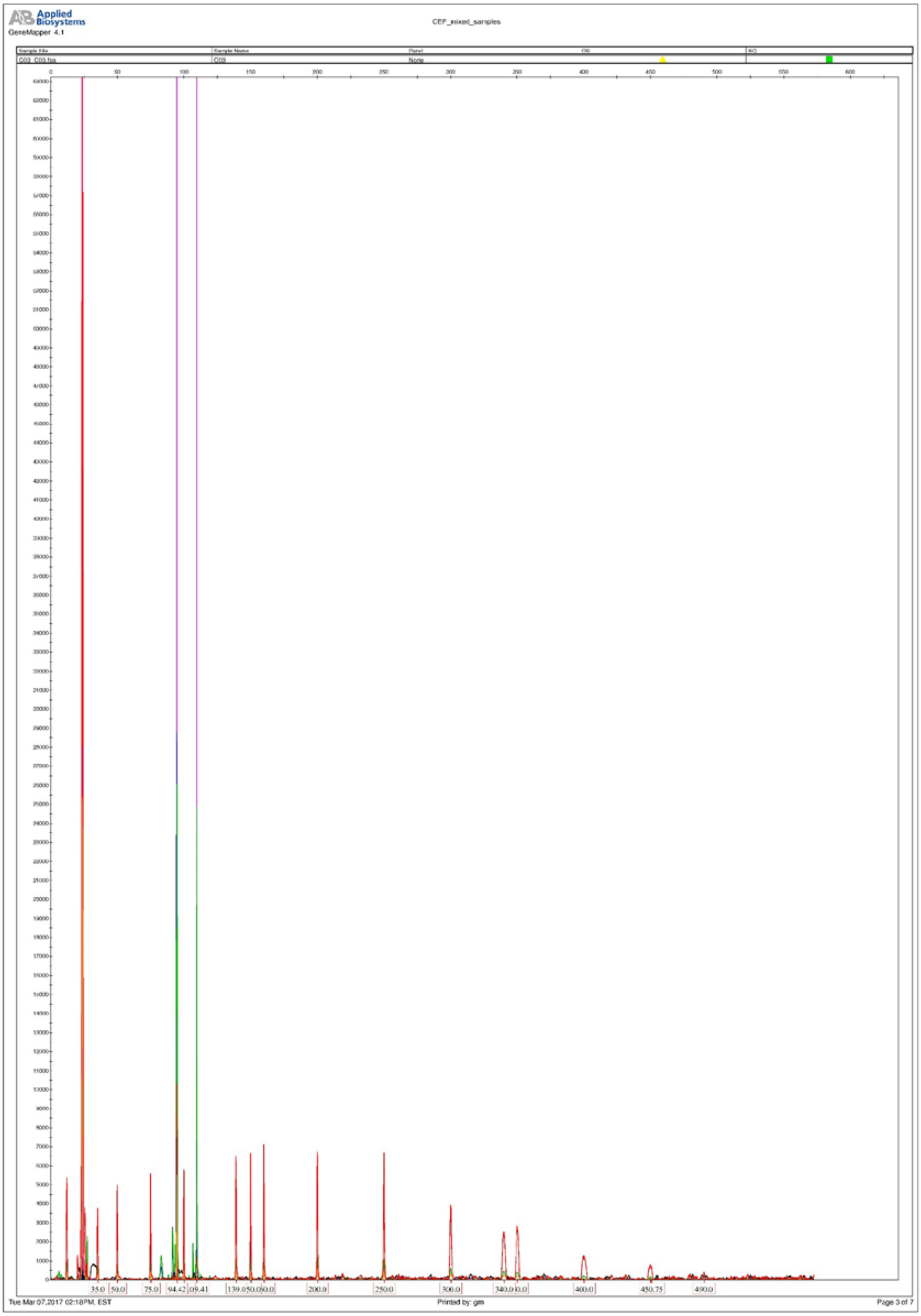

Additional file 4. Mixed Sample Electropherograms

Marker	DNA ratio 803:GB4	Microsatellite Region Sizes
**TAA87**	**50:50**	**803—109bp GB4—94bp**

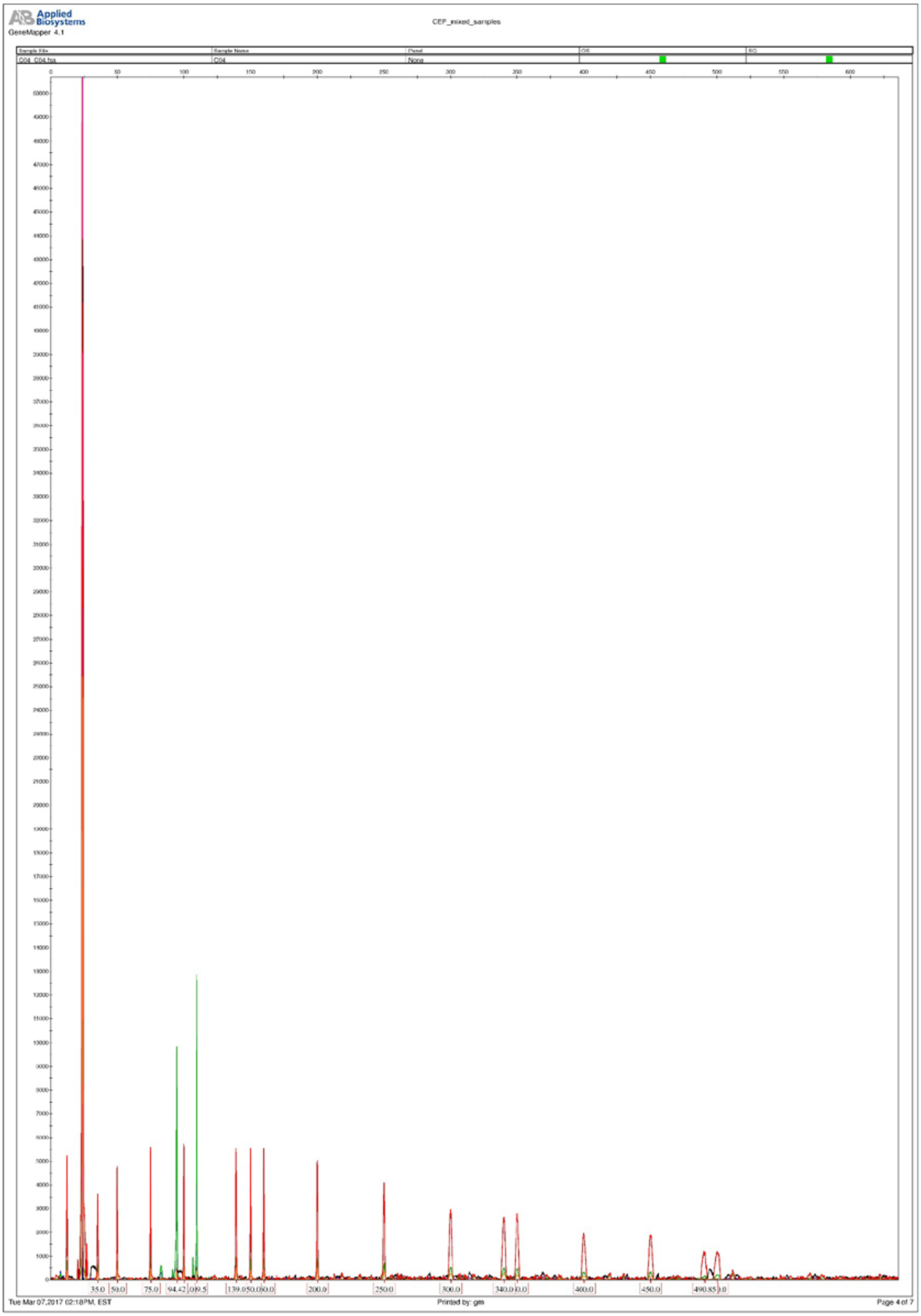

Additional file 4. Mixed Sample Electropherograms

Marker	DNA ratio 803:GB4	Microsatellite Region Sizes
**TAA87**	**80:20**	**803—109bp GB4—94bp**

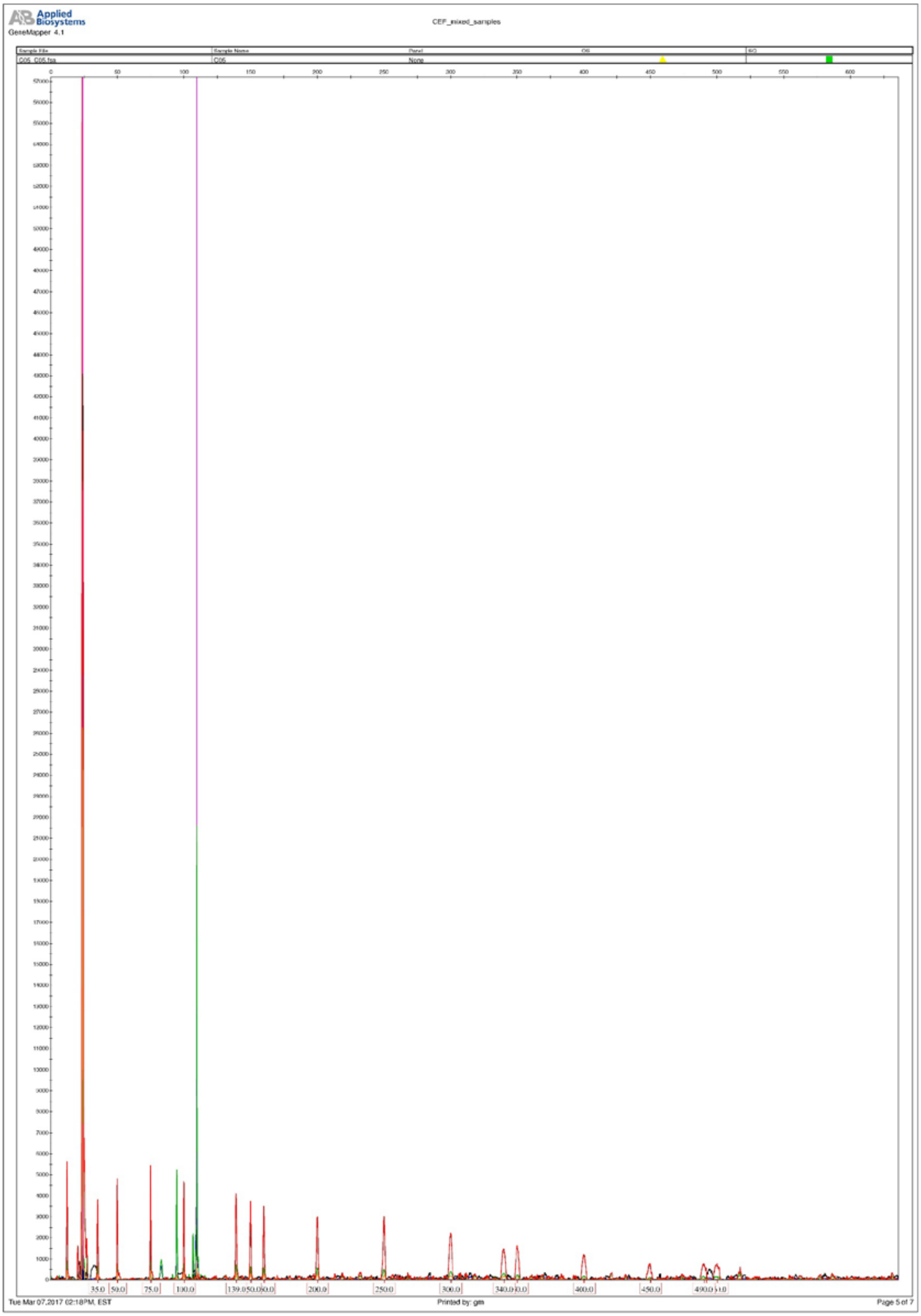

Additional file 4. Mixed Sample Electropherograms

Marker	DNA ratio 803:GB4	Microsatellite Region Sizes
**TAA87**	**90:10**	**803—109bp GB4—94bp**

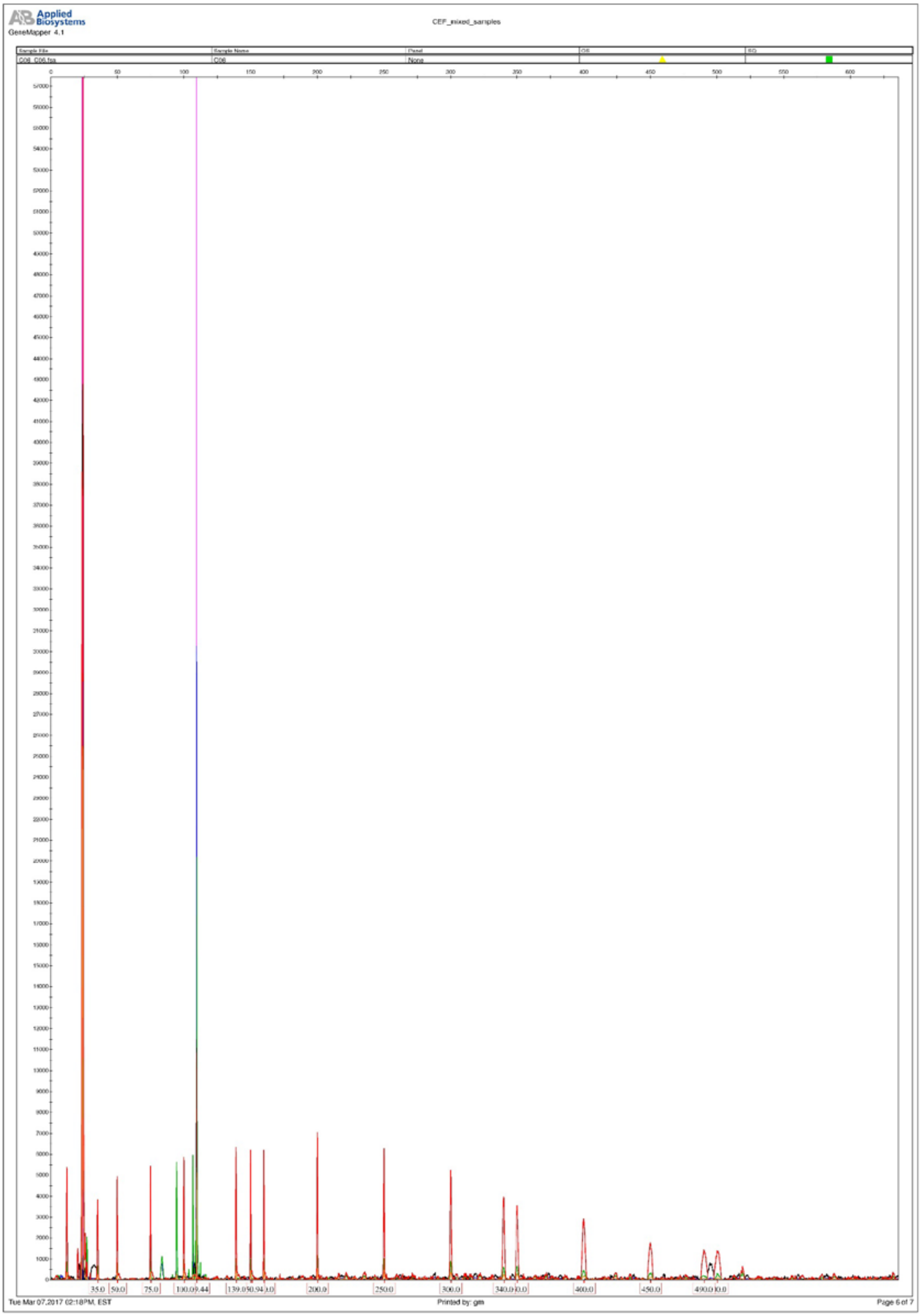

Additional file 4. Mixed Sample Electropherograms

Marker	DNA ratio 803:GB4	Microsatellite Region Sizes
**TAA87**	**95:5**	**803—109bp GB4—94bp**

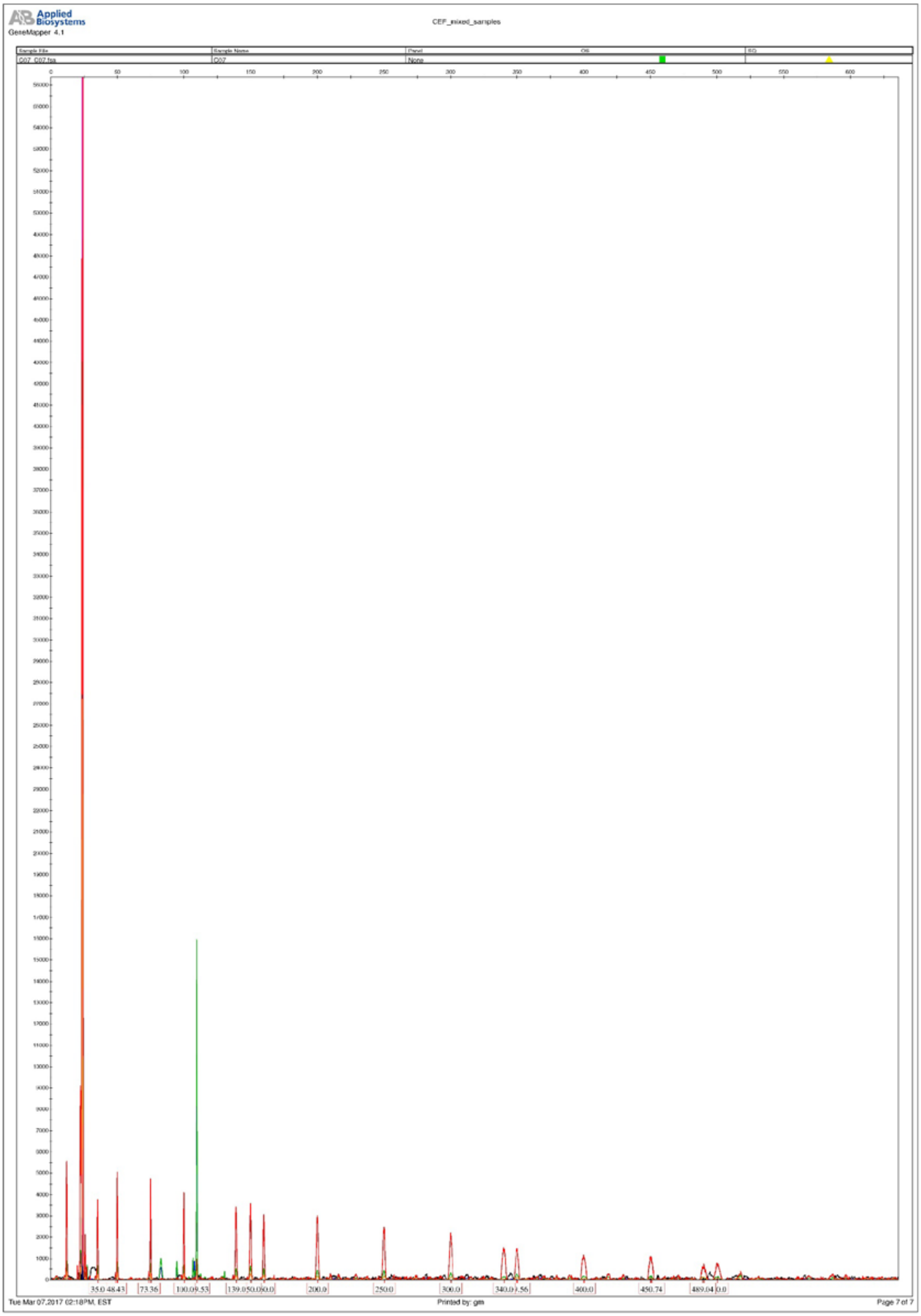

Additional file 4. Mixed Sample Electropherograms

Marker	DNA ratio 803:GB4	Microsatellite Region Sizes
**TA127**	**5:95**	**803—132bp GB4—118bp**

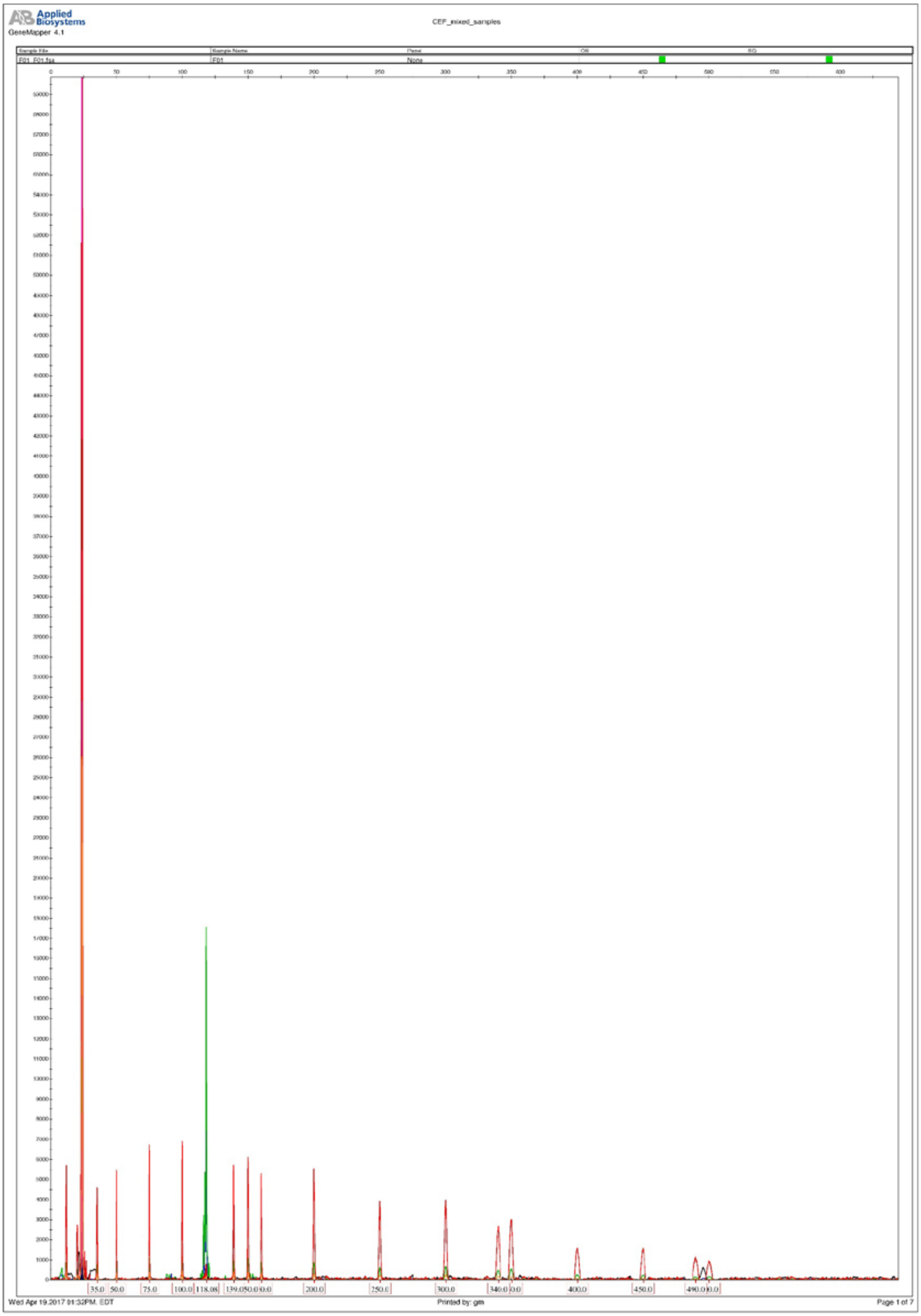

Additional file 4. Mixed Sample Electropherograms

Marker	DNA ratio 803:GB4	Microsatellite Region Sizes
**TA127**	**10:90**	**803—132bp GB4—118bp**

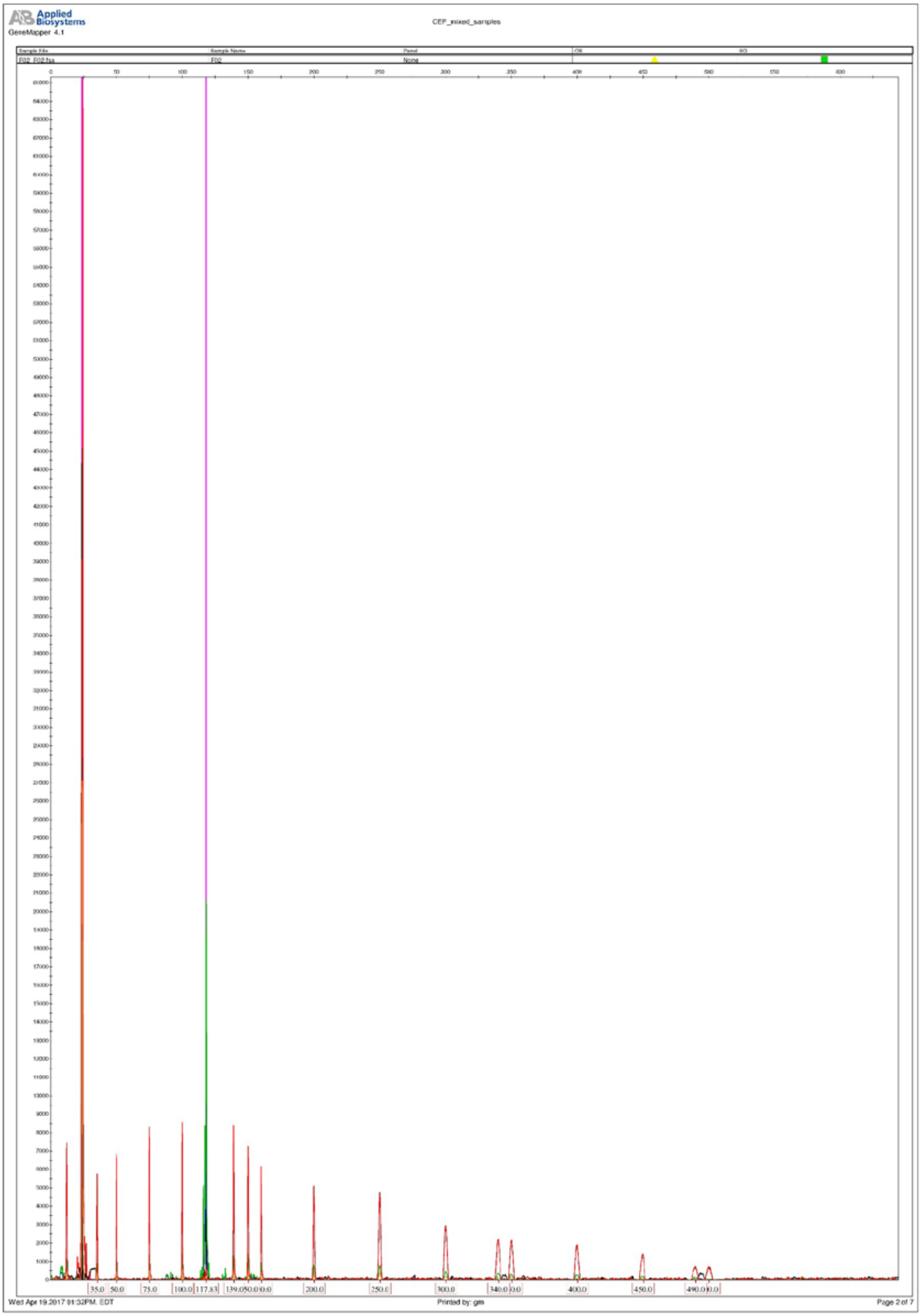

Additional file 4. Mixed Sample Electropherograms

Marker	DNA ratio 803:GB4	Microsatellite Region Sizes
**TA127**	**20:80**	**803—132bp GB4—118bp**

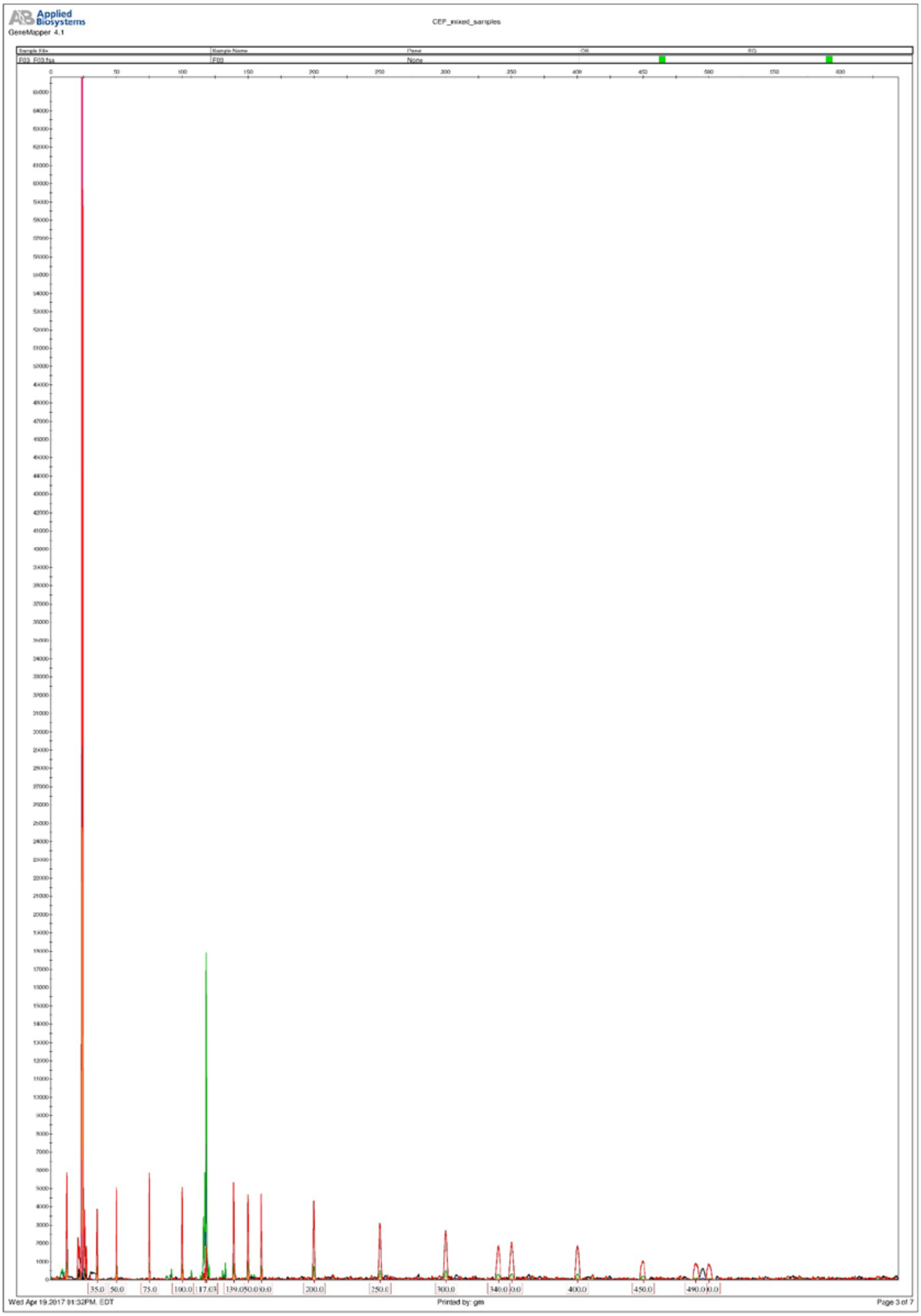

Additional file 4. Mixed Sample Electropherograms

Marker	DNA ratio 803:GB4	Microsatellite Region Sizes
**TA127**	**50:50**	**803—132bp GB4—118bp**

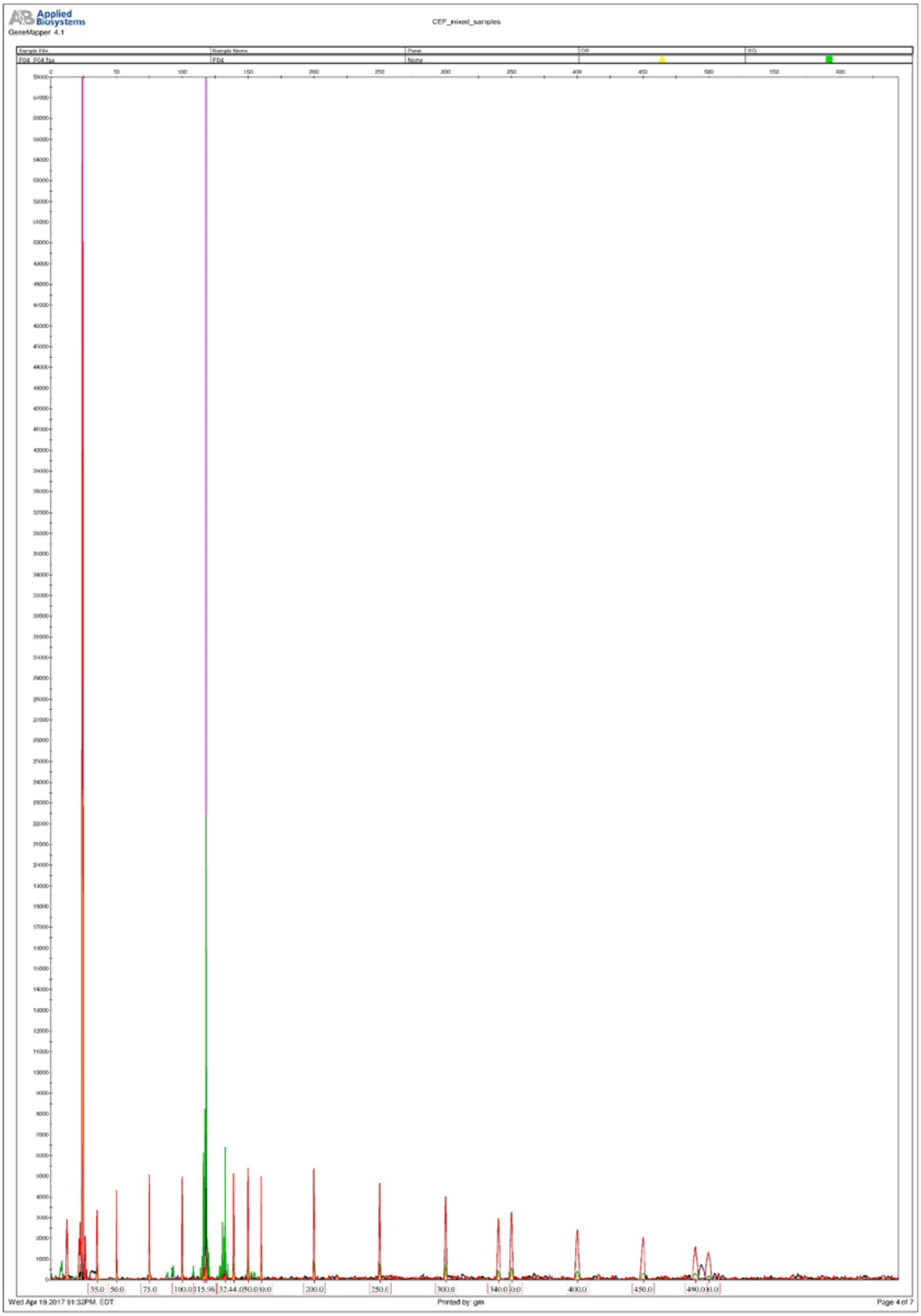

Additional file 4. Mixed Sample Electropherograms

Marker	DNA ratio 803:GB4	Microsatellite Region Sizes
**TA127**	**80:20**	**803—132bp GB4—118bp**

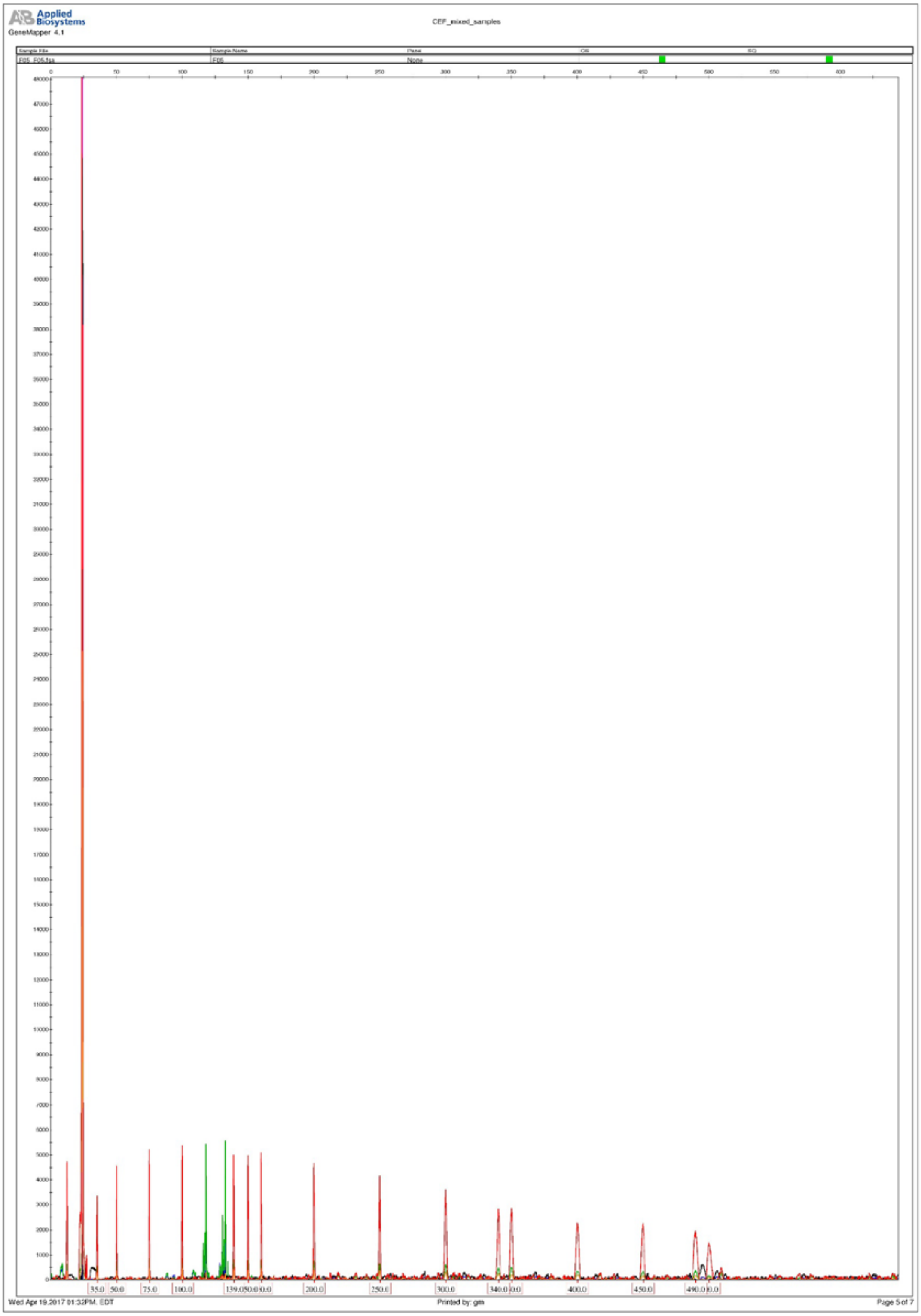

Additional file 4. Mixed Sample Electropherograms

Marker	DNA ratio 803:GB4	Microsatellite Region Sizes
**TA127**	**90:10**	**803—132bp GB4—118bp**

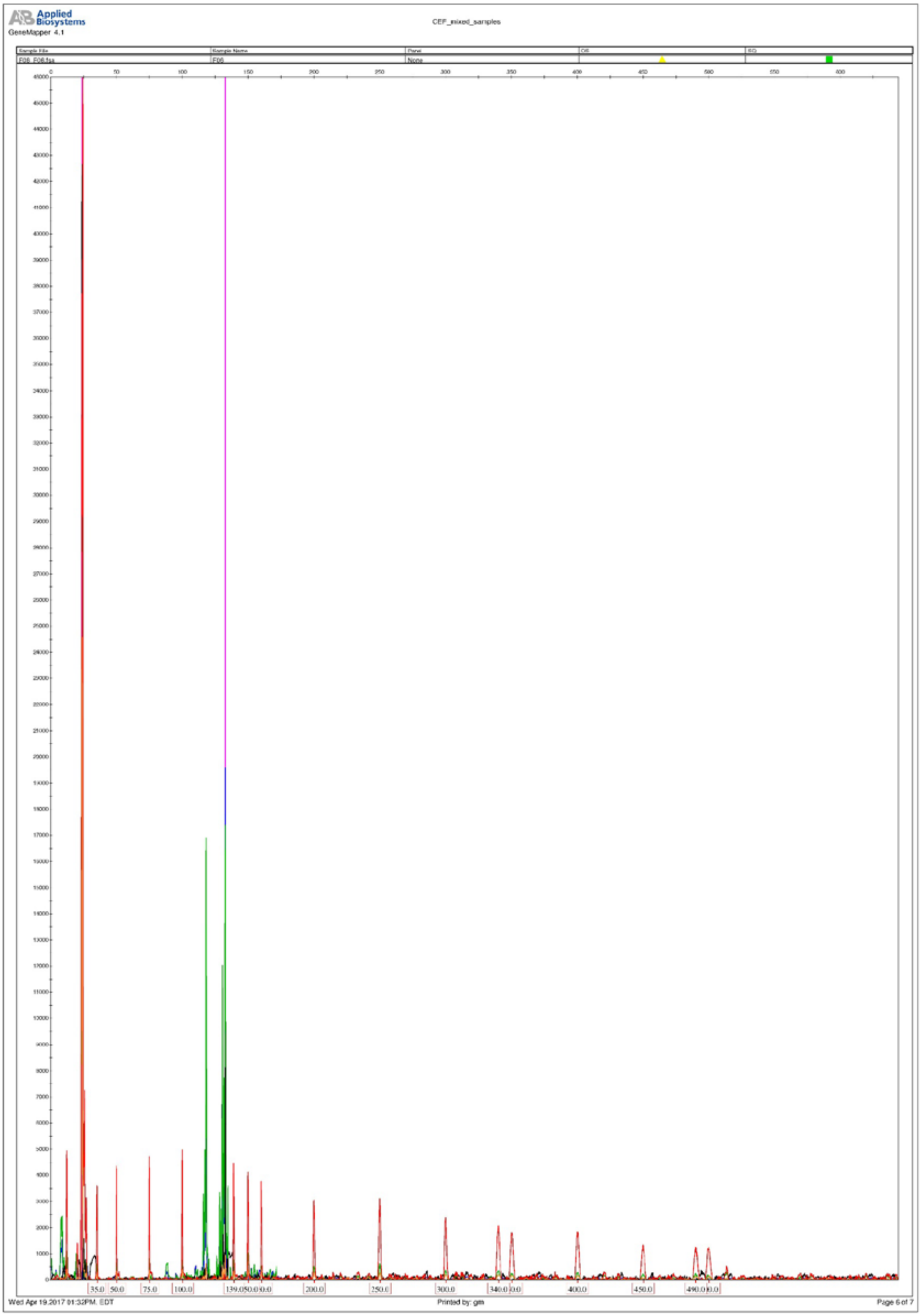

Additional file 4. Mixed Sample Electropherograms

Marker	DNA ratio 803:GB4	Microsatellite Region Sizes
**TA127**	**95:5**	**803—132bp GB4—118bp**

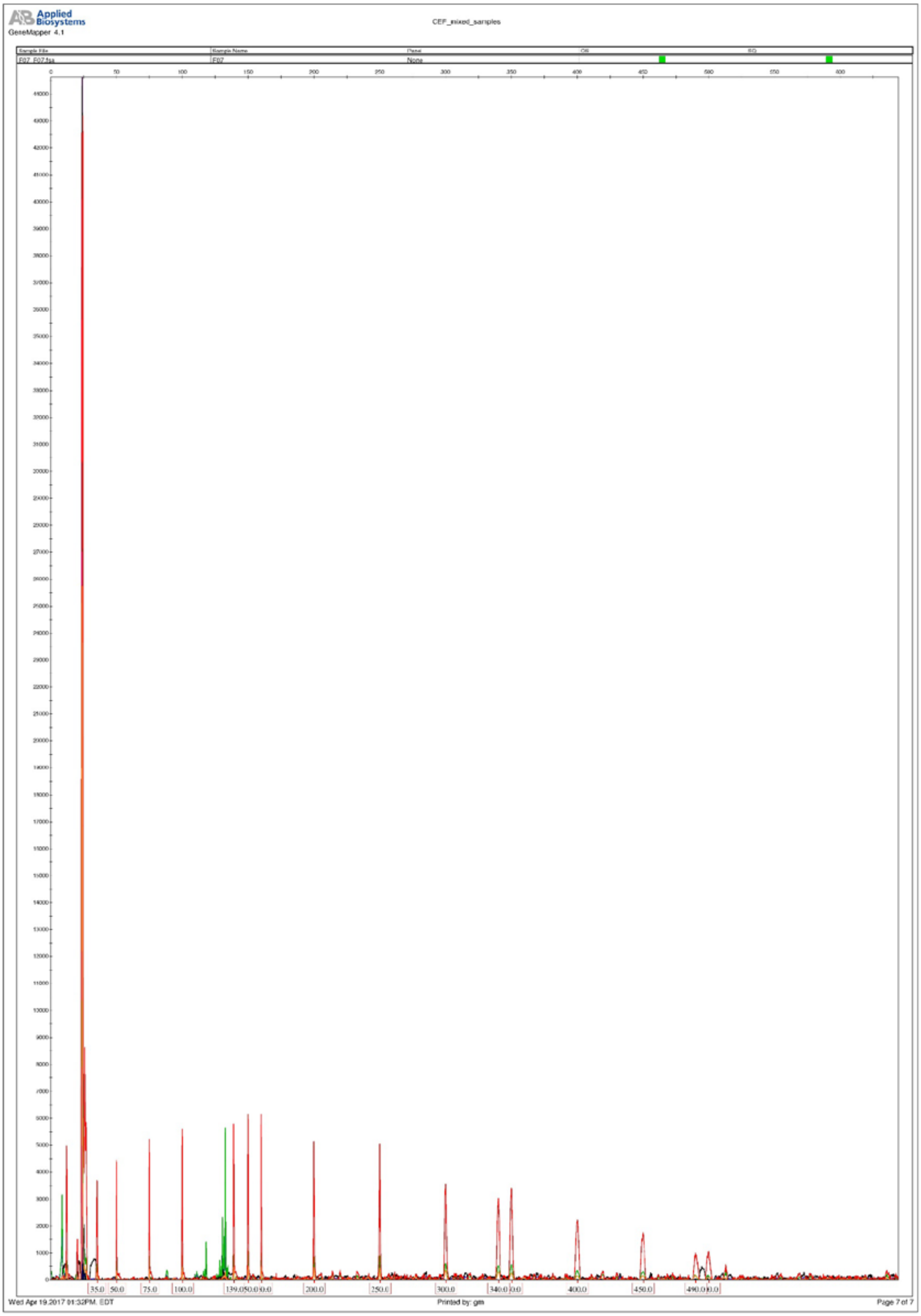

Supplement: Supplementary file 4 — Additional file 4. “Mixed Sample Electropherograms” shows representative results from mixed DNA microsatellite typing. [file 12936_2018_2210_MOESM4_ESM.pdf]
